# Post-pandemic resurgence and co-detectiondynamics of respiratory pathogens revealed by syndromic multiplex PCR

**DOI:** 10.1186/s12866-026-05081-w

**Published:** 2026-04-28

**Authors:** Ismail Selçuk Aygar

**Affiliations:** 1 Medical Microbiology Laboratory, University of Health Sciences, Etlik City Hospital, Varlık Mahallesi, Halil Sezai Erkut Street, Yenimahalle, Ankara, Türkiye; 2 Medical Microbiology Laboratory, University of Health Sciences,Gulhane Training and Research Hospital, Ankara, Türkiye

**Keywords:** Respiratory tract infections, Syndromic multiplex PCR, BioFire FilmArray

## Abstract

**Background:**

Acute respiratory tract infections are a major cause of morbidity and healthcare burden worldwide. Because many respiratory pathogens such as influenza viruses, Respiratory Syncytial Virus (RSV), Rhinovirus/Enterovirus, and SARS-CoV-2 present with overlapping clinical features, rapid and accurate etiological diagnosis is essential for effective patient management. Syndromic multiplex PCR panels, particularly the BioFire FilmArray Respiratory Panel 2.1 Plus (RP2.1 Plus), enable the simultaneous detection of a broad range of viral and bacterial pathogens, facilitating the identification of both mono- and co-infections.

**Objectives:**

This study aimed to investigate the distribution of respiratory pathogens, co-detectionpatterns, seasonal variation, and sequential infections in patients with suspected respiratory tract infections using BioFire RP2.1 Plus in a tertiary-care hospital in Türkiye.

**Methods:**

In this retrospective observational study, 360 respiratory samples obtained from 314 patients between March 2024 and March 2025 were analysed. All samples were tested using the BioFire FilmArray RP2.1 Plus system. Pathogen frequencies, mono- and co-detectionrates, demographic associations, seasonal distribution, and correlations between circulating pathogens were evaluated using appropriate statistical methods.

**Results:**

At least one respiratory pathogen was detected in 55.8% (201/360) of samples. A total of 253 pathogens were identified. The most frequent pathogen was Rhinovirus/Enterovirus (38.7%), followed by influenza A (12.3%), adenovirus (10.7%), SARS-CoV-2 (7.9%), RSV (6.3%), and *Mycoplasma pneumoniae* (4.3%). Co-detections were identified in 20.4% of positive samples, with binary co-infections being the most common. Rhinovirus/Enterovirus was the dominant pathogen in all co-detectioncategories. Women and children had significantly higher positivity rates than men and adults, respectively. Marked seasonal variation was observed: influenza A, RSV, human metapneumovirus, adenovirus, and *M. pneumoniae* were predominantly detected during the cold season (October–March), while Rhinovirus/Enterovirus circulated throughout the year. A strong positive correlation was observed between RSV and influenza A (ρ = 0.68, *p* = 0.011). Sequential sample analysis suggested that many co-detections may reflect sequential rather than simultaneous infections, potentially representing residual nucleic acid from recent infections.

**Conclusions:**

Real-life BioFire RP2.1 Plus testing demonstrated high etiological diversity, frequent co-detections, and pronounced seasonal dynamics of respiratory pathogens in the post-pandemic period, confirming the clinical and epidemiological value of syndromic multiplex PCR–based respiratory diagnostics.

## Introduction

Acute respiratory tract infections are among the leading causes of morbidity and mortality worldwide, and represent a significant burden on health systems. The differential diagnosis of pathogens such as influenza, Respiratory Syncytial Virus (RSV), Rhinovirus/Enterovirus and Severe Acute Respiratory Syndrome Coronavirus 2 (SARS-CoV-2), which present with clinically similar symptoms, cannot be reliably made based on clinical findings alone [1]. The concurrent circulation of respiratory tract pathogens has been shown to increase, with co-infections becoming more prevalent in the context of the ongoing pandemic [2–7]. This has resulted in a more complex clinical management landscape.

Consequently, the capacity to swiftly and precisely diagnose respiratory tract infections based on the causative agent has assumed paramount importance [8]. Whilst conventional techniques are both time-consuming and have limited scope, syndromic multiplex polymerase chain reaction (PCR) panels offer the advantage of detecting numerous viral and bacterial pathogens in the same sample in a short time [9–14]. The utilisation of these systems exerts a direct influence on clinical practice, encompassing the reduction of unnecessary antibiotic usage, the timely initiation of appropriate antiviral treatment, and the implementation of infection control measures in a timely manner [9, 11–13, 15].

BioFire FilmArray Respiratory Panel systems are among the most extensively utilised syndromic platforms in this domain. Over time, the scope of the pathogen coverage has been expanded, and with the addition of the SARS-CoV-2 target, the BioFire FilmArray Respiratory Panel 2.1 (RP2.1) version was developed. With the subsequent addition of Middle East Respiratory Syndrome Coronavirus (MERS-CoV), the BioFire FilmArray Respiratory Panel 2.1 Plus (RP2.1 Plus) version was developed [8]. The RP2.1 Plus panel has been demonstrated to cover a wide range of respiratory pathogens, including adenoviruses, seasonal coronaviruses, SARS-CoV-2, influenza A and B, RSV, parainfluenza viruses, Rhinovirus/Enterovirus, *Bordetella* species, and atypical bacteria [8, 16–18]. As demonstrated in clinical validation studies, this panel has been shown to exhibit high levels of both sensitivity and specificity [16, 18].

A comparison of the clinical performance of BioFire RP2.1 with that of other multiplex systems reveals that BioFire RP2.1 has a limit of detection of 50 copies/mL for SARS-CoV-2. Furthermore, it has been reported to have a clinical sensitivity of 97% and negative agreement of 100% [19]. Furthermore, these panels facilitate the reliable detection of co-infections as well as single infections. As demonstrated in previous studies, approximately one-quarter of positive samples have been shown to contain two or more pathogens. The most common causative agent identified in these co-infections is Rhinovirus/Enterovirus [20–22].

Significant changes have occurred in the epidemiological dynamics of respiratory viruses in the post-pandemic period; influenza, RSV, and other classic pathogens have re-entered circulation with marked strength [23]. This change necessitates a reassessment of current pathogen distributions and co-detectionpatterns at the regional level.

In this context, the analysis of real-life data obtained using BioFire FilmArray RP2.1 Plus offers an important opportunity to reveal the current distribution of respiratory pathogens and single and multiple infection patterns [20, 23]. The objective of this study is to make a unique contribution to the field of regional epidemiology and co-detectiondynamics. This will be achieved by analysing the results obtained with RP2.1 Plus in the Medical Microbiology Laboratory of Gülhane Training and Research Hospital.

## Materials and methods

In this retrospective observational study, multiplex PCR results of samples sent to the Medical Microbiology Laboratory of Gülhane Training and Research Hospital with suspected respiratory tract infection were examined.

Respiratory specimens were collected exclusively as nasopharyngeal swabs using sterile flocked swabs in accordance with standard clinical procedures. The swabs were then transferred directly into tubes containing viral transport medium (VTM). All samples were transported to the microbiology laboratory under cold chain conditions, stored at 2–8 °C, and processed within 24 h of collection. All samples were processed on the FilmArray^®^ 2.0 system using the BioFire FilmArray Respiratory Panel 2.1 Plus (RP2.1 Plus) kit (BioFire^®^ Diagnostics, Salt Lake City, UT, USA) in accordance with the manufacturer’s recommendations [24]. This syndromic molecular diagnostic platform automatically performs nucleic acid extraction, amplification, and multiple real-time PCR steps using closed cartridge technology. The results of these tests were reported as “detected” or “not detected” by the device and automatically transferred to the laboratory information management system (LIMS).

The RP2.1 Plus panel has been engineered to facilitate the concurrent detection and differentiation of a range of respiratory pathogens, including adenovirus, the Coronavirus 229E, HKU1, NL63 and OC43, MERS-CoV, SARS-CoV-2, human metapneumovirus, rhinovirus/enterovirus, Influenza A (A/H1, A/H3 and A/H1-2009), Influenza B, parainfluenza virus 1–4, respiratory syncytial virus, *Bordetella parapertussis*, *Bordetella pertussis*, *Chlamydia pneumoniae* and *Mycoplasma pneumoniae* nucleic acids. For each sample, the test date, patient age and gender, test result and detected pathogen(s) were obtained via a data file downloaded in Microsoft Excel format from the LIMS system.

The detection of a single pathogen in a sample is termed monoinfection, whereas the detection of two or more pathogens in the same sample is defined as co-detection, and these are classified as binary, triple, or quadruple.

Samples obtained within a 14-day period from the same patient and yielding the same pathogen result, as well as samples reported as suspicious (invalid or indeterminate), were excluded from the analysis. In temporal evaluations, months were divided into two groups: a cold period (October–March) and a warm period (April–September).

The data were collated in Microsoft Excel and analysed using SPSS v23.0 software (IBM Corp., Armonk, NY, USA). Categorical variables were presented as numbers and percentages, while continuous variables were presented as mean ± standard deviation or median (interquartile range(IQR) IQR1– IQR3) according to the distribution. Chi-square or Fisher exact tests were used for intergroup comparisons, and simultaneous circulation between pathogens was evaluated using Spearman correlation analysis. The distribution of Rhinovirus/Enterovirus in co-infections was analysed using the binomial test. The statistical significance level was accepted as *p* < 0.05, and the results were evaluated at a 95% confidence interval.

During the preparation of this article, an artificial intelligence language model was employed to refine the language, enhance the narrative, and restructure the text. The AI tool was not utilised for the generation of scientific data, the modification of results, or the interpretation of results. The authors have conducted a comprehensive review of the study’s content, verifying and approving its entirety. The scientific and ethical responsibility for the study’s findings rests solely with the authors.

This study was approved by the University of Health Sciences Gülhane Scientific Research Ethics Committee (Decision No: 2025 − 440 Decision Date: 30.09.2025). Due to the retrospective design of the study and the use of anonymized data, the requirement for informed consent to participate was waived by the University of Health Sciences Gülhane Scientific Research Ethics Committee. This applies to all patients and all patient data included in the study. The study was conducted in accordance with the Declaration of Helsinki.

## Results

The study comprised a total of 360 respiratory tract samples from 314 patients who were referred to the Medical Microbiology Laboratory of Gülhane Training and Research Hospital between March 2024 and March 2025. 69.1% of the patients were male (*n* = 217) and 30.9% were female (*n* = 97). The mean age of the overall group was 36.74 years (0–90), with a standard deviation of 27.44. IQR1 was calculated as 12, IQR3 as 61, and median 49. The mean age of female patients was 39.27 years (SD 28.44; IQR1 9, IQR3 65, median 56), while the mean age of male patients was 34.11 years (SD 26.02; IQR1 19, IQR3 60, median 41).

Of the 360 samples that were examined, 55.8% (*n* = 201) were positive for at least one respiratory pathogen, and 44.2% (*n* = 159) were negative. Following the evaluation of the 253 respiratory pathogens detected in the positive samples, it was determined that the most prevalent pathogen was Rhinovirus/Enterovirus (38.7%; *n* = 98), followed by Influenza A (12.3%; *n* = 31) and Adenovirus (10.7%; *n* = 27). SARS-CoV-2 (7.9%; *n* = 20), RSV (6.3%; *n* = 16), and *Mycoplasma pneumoniae* (4.3%; *n* = 11) were detected less frequently. The prevalence of other viral and bacterial pathogens was observed to be below 3%, and no detection was made of *Bordetella parapertussis*, *Chlamydophila pneumoniae*, and MERS-CoV. The distribution is outlined in Table 1.

**Table 1:** The distribution of respiratory tract pathogens detected in single and multiple infections

Pathogen	*n*	%
Rhinovirus/Enterovirus	98	38.74%
Influenza A	31	12.25%
Adenovirus	27	10.67%
SARS-CoV-2	20	7.91%
RSV	16	6.32%
*Mycoplasma pneumoniae*	11	4.35%
Coronavirus NL63	7	2.77%
Human metapneumovirus	7	2.77%
Coronavirus OC43	7	2.77%
Influenza B	5	1.98%
*Bordetella pertussis*	5	1.98%
Parainfluenza virus 4	5	1.98%
Parainfluenza virus 2	4	1.58%
Parainfluenza virus 3	4	1.58%
Coronavirus 229E	3	1.19%
Parainfluenza virus 1	2	0.79%
Coronavirus HKU1	1	0.40%
*Bordetella parapertussis*	0	0.00%
*Chlamydophila pneumoniae*	0	0.00%
MERS-CoV	0	0.00%
Total	253	100.00%

*SARS*-*CoV*-*2* Severe acute respiratory syndrome coronavirus 2, *MERS*-*CoV* Middle east respiratory syndrome coronavirus, *RSV* Respiratory syncytial Virus

61.2% of positive samples were obtained from adults (*n* = 123) and 38.8% from children (*n* = 78). Of the positive adults, 68.5% were male (*n* = 85) and 31.5% were female (*n* = 38), while of the positive children, 57.1% were male (*n* = 44) and 42.9% were female (*n* = 34).

The positivity rate was found to be 74.2% (*n* = 72) in female and 59.4% (*n* = 129) in male (*p* < 0.05). The positivity rate was found to be 66.1% (*n* = 78) in children and 50.8% (*n* = 123) in adults (*p* < 0.05). The mean age of positive patients was found to be 30.78 years (0–88) (SD 27.31; IQR1 9, IQR3 56, IQR 47). The mean age of positive female was 33.42 years (SD 26.58; IQR1 7, IQR3 60, IQR 53), and for positive male it was 27.26 years (SD 27.53; IQR1 9, IQR3 52, IQR 43).

The age distribution of detected pathogens was as follows: 44 (17.4%) were identified in patients aged 0–1 years, 27 (10.7%) in 1–2 years, 9 (3.6%) in 3–6 years, 25 (9.9%) in 7–17 years, 70 (27.7%) in 18–35 years, 34 (13.4%) in 36–60 years, and 44 (17.3%) in ≥ 61 years.

**Fig. 1:**
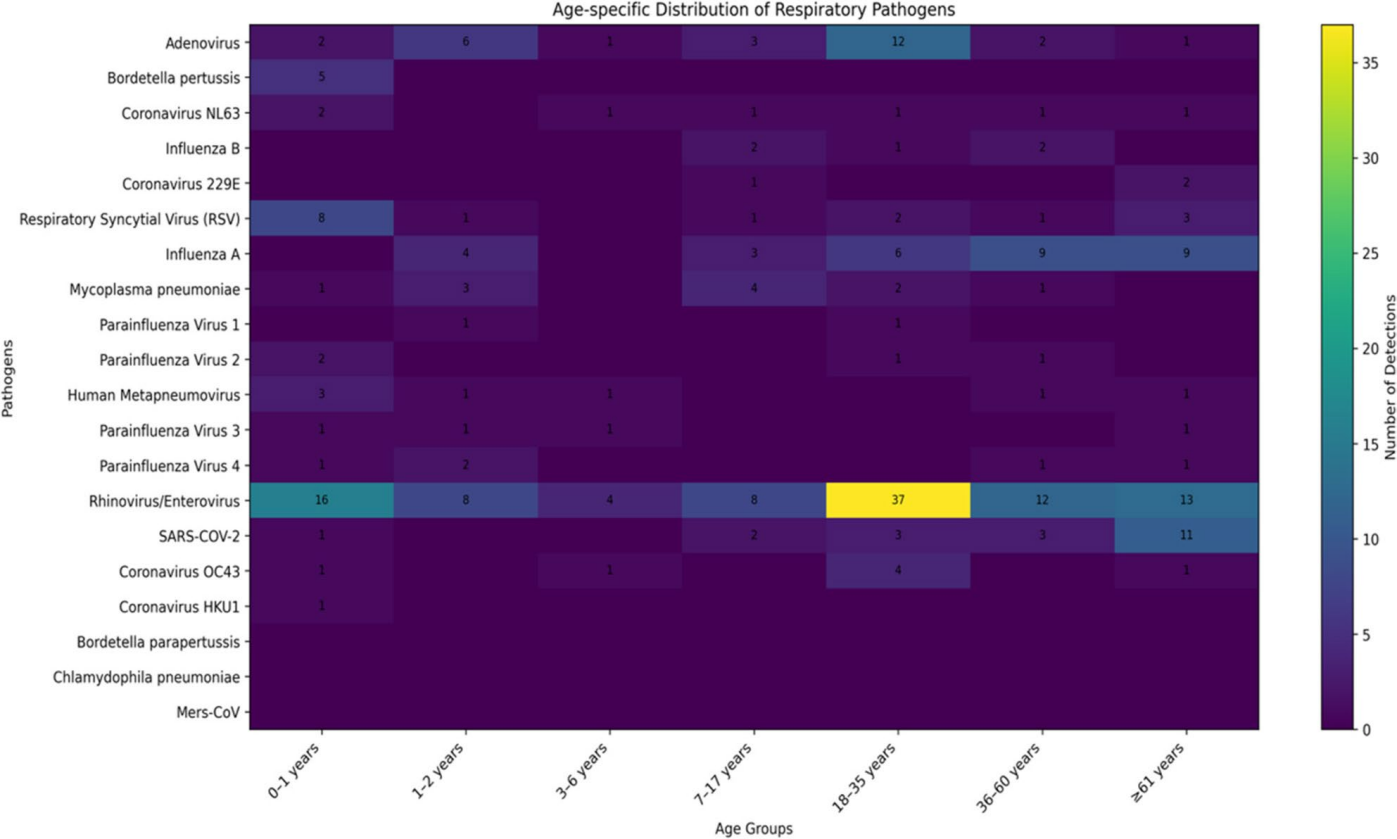
Age-specific distribution of respiratory pathogens detected by the BioFire FilmArray RP2.1 Plus panel

A subsequent age-specific analysis revealed distinct distribution patterns across respiratory pathogens. Rhinovirus/Enterovirus was the most frequently detected pathogen in all age groups, with a marked predominance in young adults (18–35 years). RSV was predominantly observed in infants (0–1 years), whereas *Mycoplasma pneumoniae* was more frequently detected in school-aged children (7–17 years). Influenza A demonstrated a marked predominance in adult demographics, particularly within the 36–60 and ≥ 61 age brackets. In a similar vein, the prevalence of SARS-CoV-2 was found to be more pronounced in patients aged ≥ 61 years, suggesting an association between age and increased susceptibility to infection. Adenovirus and other respiratory viruses were distributed across multiple age groups without a strict age-specific restriction, but demonstrated relatively higher frequencies in younger and middle-aged individuals. The distribution of respiratory pathogens across age groups was found to be statistically significant (*p* < 0.001). These findings indicate a clear epidemiological pattern that is dependent on age, and the detailed distribution is illustrated in Fig. 1.

**Fig. 2:**
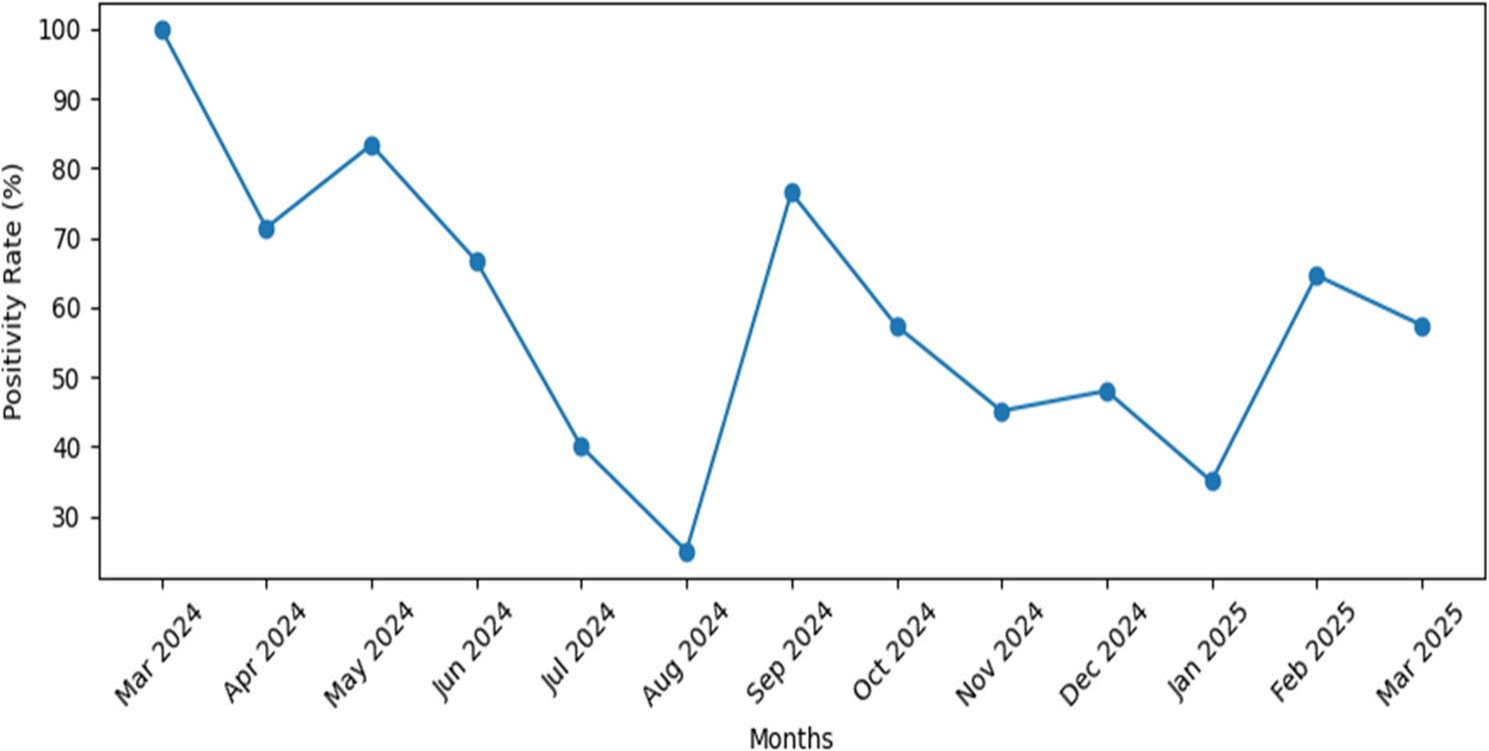
Monthly positivity rates of respiratory samples detected by the BioFire FilmArray RP2.1 Plus panel (March 2024–March 2025)

The monthly positivity rates demonstrated significant variability, with the highest values being recorded in March 2024 (100%, *n* = 13), September 2024 (76.5%, *n* = 13), and February 2025 (64.6%, *n* = 31) (*p* < 0.001). The distribution is illustrated in Fig. 2.

**Fig. 3:**
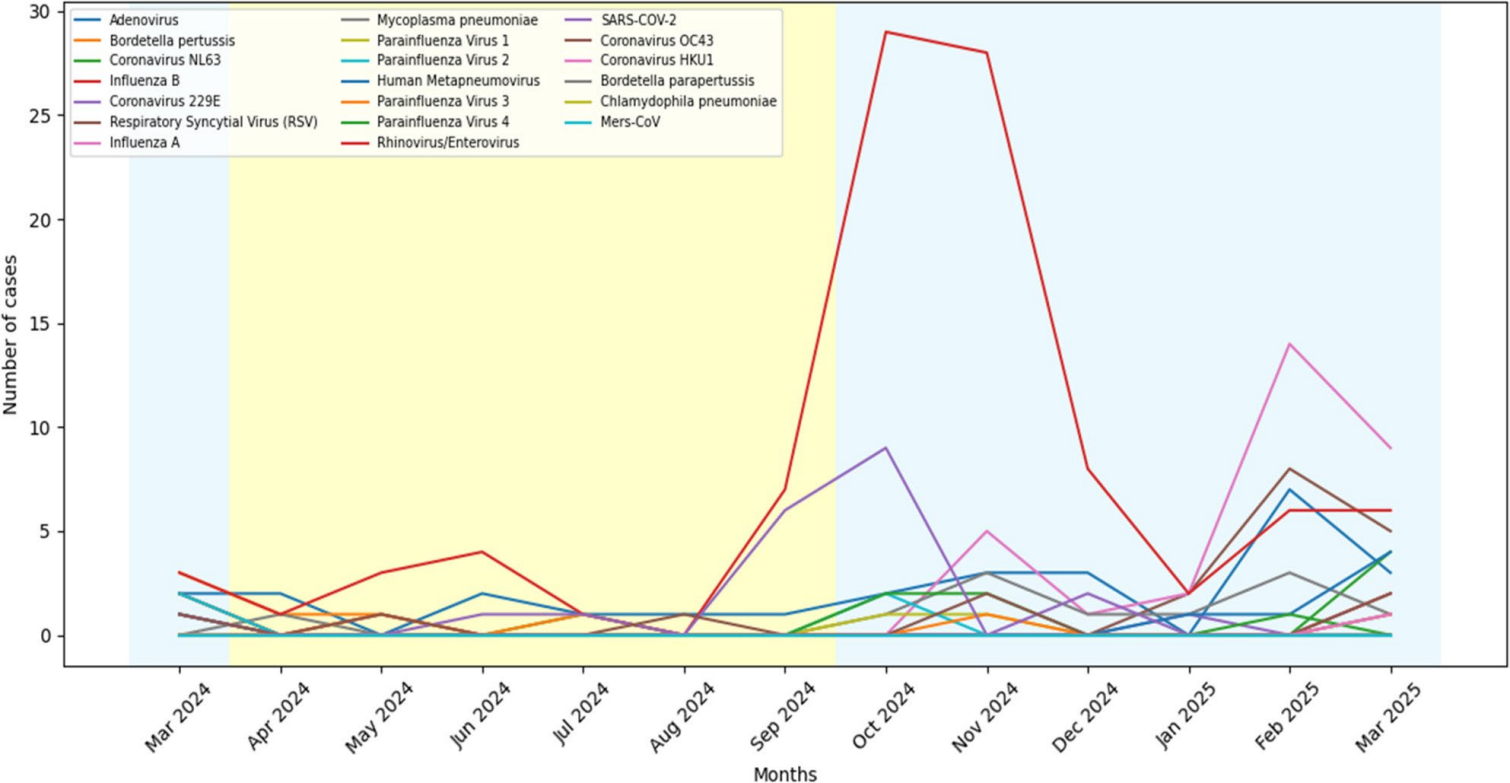
Monthly temporal patterns of respiratory pathogen detection identified by multiplex PCR (RP2.1 Plus)

A subsequent examination of the distribution of pathogens by month revealed distinct seasonal patterns. Rhinovirus/Enterovirus infections were predominantly concentrated during the cold period (October–March) (*n* = 82), while they were less frequent during the warm period (April–September) (*n* = 16). In a similar manner, Influenza A (*n* = 31), RSV (*n* = 16), and Human metapneumovirus (*n* = 7) were identified almost exclusively during the cold period, while these pathogens were not encountered during the warm period. A significant increase was also observed in Adenovirus (cold: *n* = 20, warm: *n* = 7) and *Mycoplasma pneumoniae* (cold: *n* = 10, warm: *n* = 1) during the winter months. SARS-CoV-2 was detected throughout the year, but reached higher numbers during the cold period (*n* = 12). The temporal distribution of these events is illustrated in Fig. 3.

A robust positive correlation was identified between RSV and Influenza A in the correlation analysis conducted on monthly distributions (ρ = 0.68; *p* = 0.011).

Of the 36 samples positive for Influenza, 86.1% (*n* = 31) were identified as Influenza A and 13.9% (*n* = 5) as Influenza B. Of the Influenza A positives, 58.1% (*n* = 18) were A/H3, 22.6% (*n* = 7) were A/H1-2009, and 19.3% (*n* = 6) were unsubtyped Influenza A. The vast majority of Influenza cases were detected during the cold season. Co-detection analysis demonstrated that 11.1% (*n* = 4/36) of Influenza cases were co-infected with another pathogen. The most prevalent comorbidities were identified as Influenza A + RSV (*n* = 2) and Influenza A + Rhinovirus/Enterovirus (*n* = 2).

**Fig. 4:**
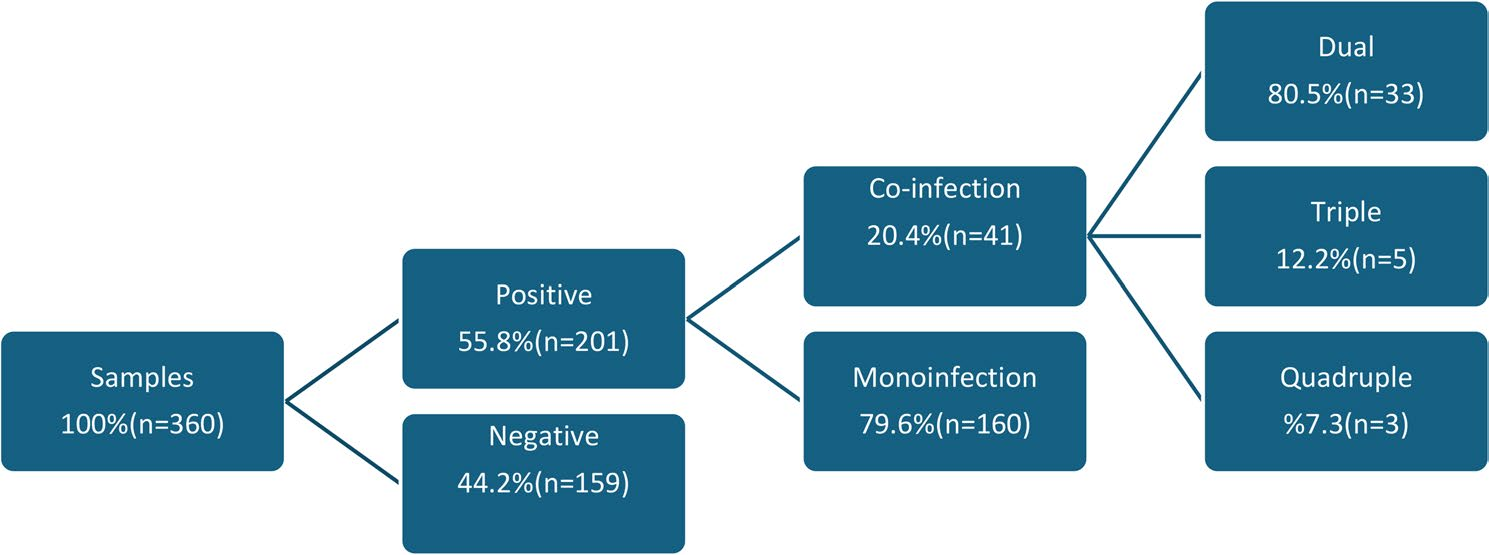
Hierarchical representation of sample positivity status and single-multiple infection distribution

In single infections, the most frequently detected pathogen was Rhinovirus/Enterovirus (*n* = 74), followed by Influenza A (*n* = 21) and SARS-CoV-2 (*n* = 15). A total of 33 double, 5 triple, and 3 quadruple co-detections were identified (Fig. 4).

A comprehensive analysis of co-detections was performed to characterise the combinations, frequency, distribution, and dynamics of co-detected pathogens. Binary co-detections were the most frequent pattern (*n* = 33), followed by triple (*n* = 5) and quadruple co-detections (*n* = 3). Detailed combinations and their exact frequencies are presented in Table 2.

**Table 2:** Detailed combinations, frequency, and distribution of respiratory pathogen co-detections

Co-detection Type	Combination	Frequency(*n*)
Binary	Coronavirus NL63 + Human Metapneumovirus	1
	Human Metapneumovirus + Respiratory Syncytial Virus (RSV)	1
	Adenovirus + Rhinovirus/Enterovirus	8
	Human Metapneumovirus + Influenza A	1
	Adenovirus + Human Metapneumovirus	1
	Coronavirus NL63 + Rhinovirus/Enterovirus	1
	Influenza A + Respiratory Syncytial Virus (RSV)	2
	Influenza A + Rhinovirus/Enterovirus	2
	*Mycoplasma pneumoniae + Rhinovirus/Enterovirus*	2
	Adenovirus + Influenza A	2
	*Mycoplasma pneumoniae* + Respiratory Syncytial Virus (RSV)	1
	Coronavirus 229E + Human Metapneumovirus	1
	Rhinovirus/Enterovirus + SARS-COV-2	3
	Coronavirus OC43 + Rhinovirus/Enterovirus	1
	Parainfluenza Virus 4 + Rhinovirus/Enterovirus	1
	Adenovirus + Parainfluenza Virus 1	1
	Parainfluenza Virus 2 + Rhinovirus/Enterovirus	1
	Adenovirus + SARS-COV-2	1
	*Bordetella pertussis* + Rhinovirus/Enterovirus	1
	*Bordetella pertussis* + Coronavirus NL63	1
Triple	Coronavirus 229E + Influenza A + Respiratory Syncytial Virus (RSV)	1
	Coronavirus OC43 + *Mycoplasma pneumoniae* + Rhinovirus/Enterovirus	1
	Adenovirus + Rhinovirus/Enterovirus + SARS-COV-2	1
	*Mycoplasma pneumoniae* + Parainfluenza Virus 1 + Parainfluenza Virus 4	1
	*Bordetella pertussis* + Parainfluenza Virus 3 + Rhinovirus/Enterovirus	1
Quadruple	Coronavirus NL63 + Coronavirus OC43 + Influenza A + Respiratory Syncytial Virus (RSV)	1
	Adenovirus + Influenza A + Parainfluenza Virus 4 + Rhinovirus/Enterovirus	1
	*Bordetella pertussis* + Coronavirus NL63 + Coronavirus OC43 + Human Metapneumovirus	1
Total		41

Among binary co-detections, Rhinovirus/Enterovirus was the most commonly involved pathogen, frequently co-detected with Adenovirus, Influenza A, *Mycoplasma pneumoniae*, and SARS-CoV-2. Triple and quadruple co-detections were less frequent and demonstrated heterogeneous patterns involving both viral and bacterial pathogens.

The distribution of co-detections according to sex demonstrated a predominance in male patients, with 23 binary, 4 triple, and 3 quadruple co-detections observed in males compared to 10 binary and 1 triple co-detections in females.

The age distribution revealed that co-detections occurred across a wide age range (binary: 0–79 years; triple: 0–63 years; quadruple: 0–25 years). The mean age was 21.8 years for binary, 21.0 years for triple, and 9.0 years for quadruple co-detections, suggesting a shift toward younger age groups with increasing co-detection complexity.

Rhinovirus/Enterovirus was identified in 60.6% (*n* = 20/33) of binary co-detections, 60% (*n* = 3/5) of triple co-detections, and 33% (*n* = 1/3) of quadruple co-detections. The binomial test revealed that these rates did not significantly exceed the expected random distribution (*p* > 0.05). However, Rhinovirus/Enterovirus remained the most prevalent pathogen across all co-detection types.

In comparison with single infections, it was observed that certain pathogens demonstrated a heightened propensity for co-occurrence. Furthermore, 40% (8/20) of Adenovirus cases, 25% (2/8) of *Mycoplasma pneumoniae* cases, 16.7% (3/18) of SARS-CoV-2 cases, and 8.7% (2/23) of Influenza A cases were found to be associated with rhinovirus/enterovirus. Furthermore, an association between Influenza A and RSV was observed in 8.7% (2/23) of cases.

**Fig. 5:**
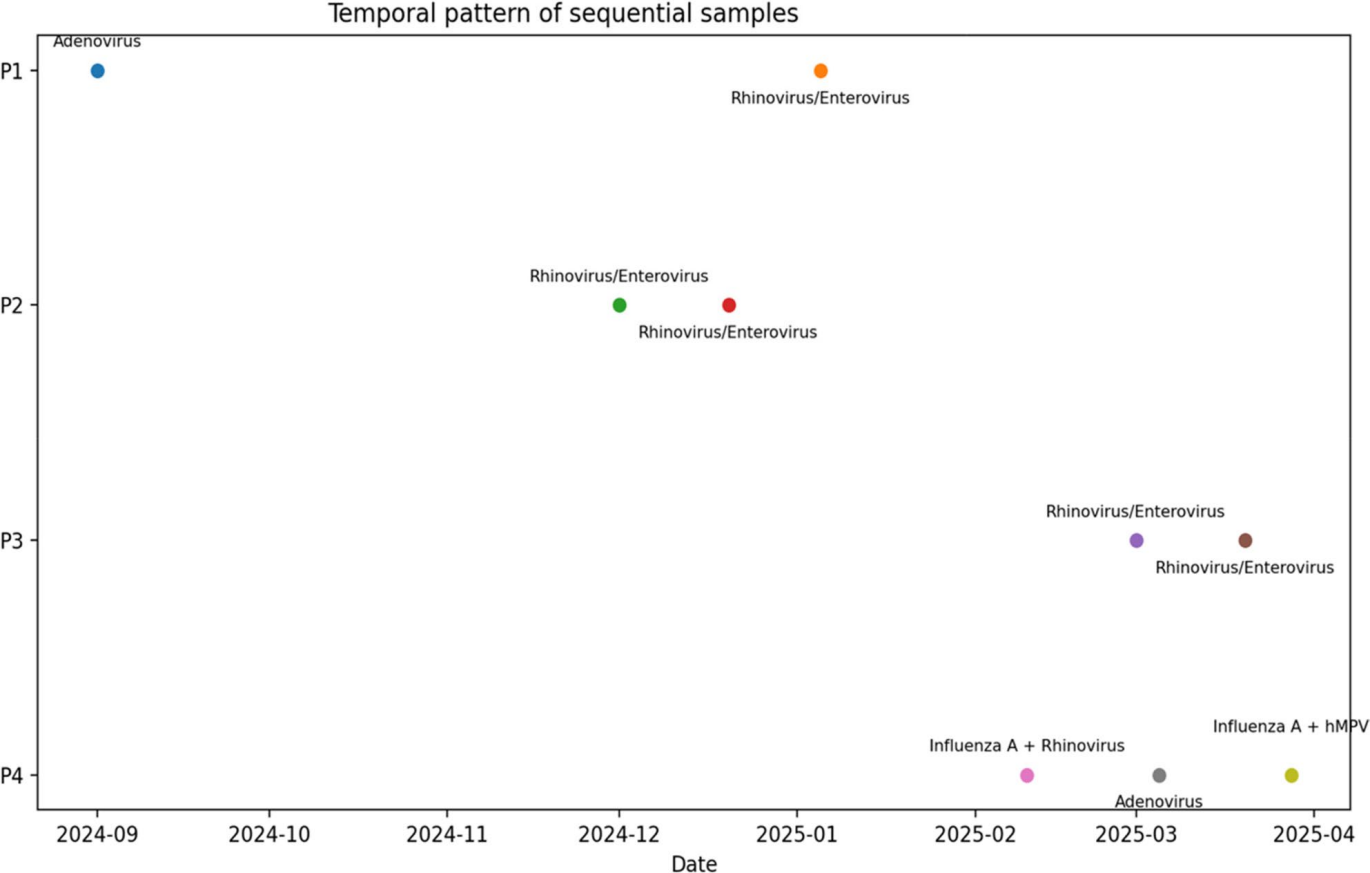
The temporal distribution of pathogens detected in consecutive respiratory tract samples from the same patients. P: Patient, hMPV: Human Metapneumovirus

A subsequent examination of the temporal distribution of samples obtained from the same patient at different times revealed distinct patterns of repeated and sequential infections (Fig. 5).

## Discussion

In this study, the distribution of pathogens, co-detectionpatterns, seasonality, and sequential sample patterns in respiratory tract samples were analysed using BioFire FilmArray RP2.1 Plus. The findings of the present study demonstrate that respiratory tract pathogens exhibit high etiological diversity, widespread co-infection, and significant seasonal fluctuations in the post-pandemic period.

In the present study, Rhinovirus/Enterovirus constituted 38.7% (*n* = 98/253) of the total positive pathogens, and was identified as the dominant pathogen in both monoinfections and co-infections. The year-round detection of Rhinovirus/Enterovirus and its more limited seasonal fluctuation compared to Influenza and RSV are consistent with the pattern reported in syndromic panel-based epidemiological studies [25, 26]. Multicentre analyses utilising BioFire RP2.1 have demonstrated that Rhinovirus/Enterovirus is the most prevalent component of co-infections, manifesting particularly in conjunction with adenovirus, RSV and SARS-CoV-2 [19].

It is an established fact that respiratory tract infections demonstrate a well-defined age-dependent distribution, with specific pathogens predominating in distinct age groups. It has been reported in numerous studies that viral respiratory infections are more prevalent in paediatric populations. Conversely, certain pathogens, including influenza viruses and SARS-CoV-2, are more frequently detected in adults [25, 26]. RSV has been identified as a leading pathogen in infants and young children, largely due to immature immune responses and smaller airway anatomy, which increase susceptibility to lower respiratory tract involvement [27, 28]. In contrast, *Mycoplasma pneumoniae* infections are more commonly observed in school-aged children and adolescents, likely reflecting increased exposure in school environments and differences in host immune responses [29, 30].

Rhinovirus, a virus that is prevalent in the global population, has been reported in all age groups. However, it is particularly common in adults, where repeated exposure and partial immunity may lead to frequent but generally milder infections [12, 31]. In a similar manner, influenza viruses have been demonstrated to exhibit elevated detection rates within adult and elderly populations, a phenomenon that is partly attributable to waning immunity and comorbid conditions that augment susceptibility to infection and symptomatic disease [31, 32]. It has been demonstrated that the novel severe acute respiratory syndrome (SARS-CoV-2) is associated with a disproportionate impact on adult populations, particularly those of advanced age, exhibiting elevated detection rates and more severe clinical outcomes in comparison to children [31, 33].

The present study demonstrated a statistically significant association between age groups and the distribution of pathogens (*p* < 0.001). In accordance with the extant literature, RSV was predominantly observed in infants, whereas *Mycoplasma pneumoniae* was more frequently detected in school-aged children. Rhinovirus/enterovirus was the most prevalent pathogen across all age groups, with a marked predominance in young adults, while influenza A and SARS-CoV-2 were more frequently identified in older individuals. These findings serve to reinforce the notion that age-related differences in immune system maturity, exposure patterns, and pathogen-specific susceptibility play a pivotal role in shaping the epidemiology of respiratory infections.

The concordance between our findings and those of previous studies, including those conducted in different geographic regions and during different epidemiological periods, suggests that age-dependent distribution patterns of respiratory pathogens are highly consistent and reproducible [34, 35]. These patterns may also be indicative of disparities in social behaviour, vaccination coverage, and prior immunity across age demographics. Taken together, these findings highlight the importance of age-stratified diagnostic and therapeutic strategies and support the clinical utility of multiplex PCR panels in optimising the management of respiratory tract infections.

Our seasonal analyses revealed a significant concentration of Influenza A, RSV, and Human Metapneumovirus during the cold period (October–March). This finding is consistent with syndromic panel data indicating that Influenza and RSV peak during the winter months [36, 37]. In contrast, the relatively stable detection of Rhinovirus/Enterovirus throughout the year suggests that this pathogen is constantly circulating within the community [36, 37]. A robust positive correlation was identified between RSV and Influenza A in the monthly distribution analysis. This finding reflects a shared seasonal pattern, with both pathogens increasing during the cold period (October–March). However, it is important to note that this correlation represents a population-level temporal trend rather than a high frequency of simultaneous co-detection at the individual patient level. A limited number of RSV-Influenza A co-detections were observed in the dataset, indicating that these viruses do not commonly occur together within the same patient despite their concurrent seasonal peaks.

Co-detections were identified in 20.4% (*n* = 41/201) of positive samples in the present study. The co-detection rate reported in comparative studies using BioFire RP2.1 is approximately 26%, suggesting that the findings of this study are consistent with the extant literature [19]. Furthermore, the observation that two-agent co-infections are more prevalent than three- and four-agent co-infections is consistent with the distribution reported in syndromic PCR series [38, 39].

The predominance of Rhinovirus/Enterovirus in co-infections is also biologically significant. Research has demonstrated that the replication of other respiratory viruses, notably SARS-CoV-2, can be suppressed in the presence of rhinovirus, thereby Influencing infection patterns in a manner contingent on viral interactions [39]. Moreover, FilmArray-based clinical studies have reported that viral infections increase susceptibility to secondary pathogens by disrupting the epithelial barrier [40, 41]. In addition to these findings, a detailed analysis of co-detection patterns revealed that binary co-detections were the most frequent, followed by triple and quadruple combinations. This distribution is consistent with previous multiplex PCR-based studies, which have reported that most multiple detections involve only two pathogens, whereas higher-order co-detections are relatively uncommon and heterogeneous [32, 42]. In our cohort, Rhinovirus/Enterovirus was the most frequently involved pathogen across co-detection groups, often in combination with Adenovirus, Influenza A, and other respiratory viruses. Similar observations have been reported in previous studies, where Rhinovirus/Enterovirus was identified as a dominant pathogen in co-detection patterns, likely due to its high prevalence and prolonged persistence in the respiratory tract [25, 43].

When the distribution of co-detections was evaluated, higher-order co-detections tended to occur in younger patients, and a slight predominance was observed in male individuals. These findings are in line with previous reports indicating that younger age groups are more likely to exhibit multiple pathogen detections, possibly due to increased exposure and differences in immune response [44].

Furthermore, the observed distribution of binary and higher-order co-detections provides insight into co-detection dynamics, indicating that simpler co-detection patterns are more common, whereas complex multi-pathogen detections occur less frequently. Previous multiplex panel studies have similarly suggested that most co-detections reflect overlapping circulation of respiratory pathogens rather than stable or biologically synergistic interactions between multiple pathogens [33, 45].

The detection of SARS-CoV-2 throughout the year, albeit with a higher prevalence during the colder months, suggests that this pathogen has now adopted a seasonal profile similar to that of classic respiratory viruses. Research employing RP2.1 and RP2.1 Plus has indicated that the presence of SARS-CoV-2 is observed more frequently during the same periods as Influenza and RSV [17, 19].

As demonstrated in Fig. 4, sequential sample analyses indicate that a considerable proportion of co-detections may be attributable to sequential infection processes rather than simultaneous exposure. It should be noted that multiplex PCR assays detect nucleic acids that may persist after the resolution of active infection. Therefore, co-detections observed in this study may not necessarily represent simultaneous active infections but may instead reflect residual nucleic acid from recent sequential infections. The re-detection of Rhinovirus/Enterovirus in some patients within weeks and its temporal replacement with different viral pathogens suggests that the respiratory ecosystem can undergo changes over a long period after viral infection. The pivotal function of viral interactions and interferon responses in this process has been previously examined in syndromic panel studies [46, 47].

The findings demonstrate the potential of syndromic multiplex PCR panels to facilitate not only the detection of single pathogens but also the elucidation of co-infections and temporal infection dynamics. The comprehensive range of pathogens covered by the assay, coupled with its exceptional analytical performance, serves to reinforce the efficacy of pathogen-based diagnosis, particularly during the winter months when multiple respiratory pathogens, including Influenza, RSV, and SARS-CoV-2, are in circulation [17, 19, 20, 46, 48].

The pathogen distribution and epidemiological patterns observed in this study are largely consistent with findings from different regions of Türkiye, although regional variations are evident. Studies from Istanbul have reported Rhinovirus/Enterovirus and influenza viruses as the most prevalent pathogens with marked seasonal variation [42, 49]. Similarly, data from eastern Türkiye (Kars) indicate a predominance of Influenza A and B, whereas a study from Kahramanmaraş demonstrated a higher contribution of bacterial pathogens, particularly Haemophilus influenzae and Streptococcus pneumoniae, highlighting regional differences in pathogen spectrum [50, 51] .

The frequency and structure of co-detections in our study are also in line with national data, where co-detection rates remain relatively low and are predominantly limited to binary combinations [50, 51]. In addition, the frequent involvement of Rhinovirus/Enterovirus in co-detections is consistent with previous reports, likely reflecting its high prevalence and prolonged persistence in the respiratory tract [42 ,50].

Overall, these findings indicate that while respiratory pathogen circulation in Türkiye follows common seasonal trends, regional differences in dominant pathogens and co-detection profiles emphasize the importance of local epidemiological surveillance.

The study is not without its limitations. The analyses were conducted within the confines of the laboratory, and the clinical severity, duration of hospitalisation, or alterations in treatment regimens were not subjects of evaluation. Moreover, the absence of confirmatory culture or viral load measurement limits a more detailed interpretation of the clinical significance of some pathogens. However, the high analytical performance of RP2.1 Plus and the utilisation of real-life data suggest that regional respiratory epidemiology is reliably reflected [17, 19, 20, 46, 48].

A fundamental limitation of molecular diagnostic methods is that PCR-based assays detect nucleic acids and cannot distinguish between viable, actively replicating pathogens and residual nucleic acid from a past infection. Consequently, the co-detections observed in this study may not always represent simultaneous active infections, but rather may be indicative of residual genetic material from recent or sequential infections. This limitation must be considered when interpreting co-detection patterns and the hypothesis of sequential infections.

Furthermore, the study was based on retrospective data obtained from a single tertiary-care hospital in Ankara, which may limit the generalisability of the findings to other regions or the broader population. The confirmation of these epidemiological patterns would be aided by multicentre studies incorporating diverse populations. Moreover, the absence of pre-pandemic data generated using the same diagnostic platform limits direct temporal comparisons. Following the implementation of the syndromic multiplex PCR panel in our laboratory in 2024, no comparable pre-pandemic dataset is available for analysis. It is recommended that future studies incorporate pre-pandemic datasets, utilising comparable methodologies, in order to facilitate more robust temporal comparisons.

In conclusion, the present study revealed that respiratory pathogens in the post-pandemic period exhibit high diversity, widespread co-infection, and dynamic seasonality. The predominant role of Rhinovirus/Enterovirus in both single and multiple infections, the substantial concentration of Influenza and RSV in colder months, and the seasonal profile of SARS-CoV-2 provide robust support for the diagnostic and epidemiological value of syndromic multiplex PCR-based approaches.

## Data Availability

All data generated or analysed during this study are included in this published article.

## Abbreviations

RSV: Respiratory Syncytial Virus
SARS-CoV-2: Severe Acute Respiratory Syndrome Coronavirus 2
MERS-CoV: Middle East Respiratory Syndrome Coronavirus
RP2.1: BioFire FilmArray Respiratory Panel 2.1
VTM: Viral transport medium
RP2.1: Plus BioFire FilmArray Respiratory Panel 2.1 Plus
PCR: Polymerase Chain Reaction
LIMS: Laboratory Information Management System
IQR: Interquartile Range
hMPV: Human Metapneumovirus
